# Breast cancer subtypes predict the preferential site of distant metastases: a SEER based study

**DOI:** 10.18632/oncotarget.15856

**Published:** 2017-03-02

**Authors:** Qi Wu, Juanjuan Li, Shan Zhu, Juan Wu, Chuang Chen, Qian Liu, Wen Wei, Yimin Zhang, Shengrong Sun

**Affiliations:** ^1^ Department of Breast and Thyroid Surgery, Renmin Hospital of Wuhan University, Wuhan, Hubei, P. R. China; ^2^ Department of Pathology, Renmin Hospital of Wuhan University, Wuhan, Hubei, P. R. China

**Keywords:** breast cancer subtypes, distant metastases, SEER

## Abstract

**Background and Aims:**

This study aimed to access possible relationships between breast cancer subtypes and sites of distant metastasis in breast cancer.

**Results:**

A total of 243,896 patients, including 226,451 cases in control groups were identified. Bone metastasis was found in 8848 cases, compared with 1,000 brain metastasis cases, 3434 liver metastasis cases and 4167 lung metastasis cases. Patients with all subtypes were most prone to bone metastases, the incidence of bone metastasis in HR+/HER2+ subtype was up to 5.1 %. Further, HR−/HER2+ subtype patients had a higher probability of brain (OR = 1.978) metastasis compared to HR+/HER2− subtype patients. In addition, liver metastasis was more frequently observed in the HER2 positive subtypes compared with HER2 negative subtypes. Patients with TN primarily presented lung metastasis, but it made no difference in the probability of lung metastases of all subtypes.

**Materials and Methods:**

Using the 2010–2013 Surveillance, Epidemiology, and End Results Program(SEER) data, a retrospective, population-based cohort study to investigate tumor subtypes-specific differences in the sites of distant metastasis. Metastatic patterns information was provided for bone, brain, liver and lung. The breast cancer was classified into four subtypes: hormone receptor (HR) +/ human epidermal growth factor receptor 2 (HER2) −, HR+/HER2+, HR−/HER2+ and triple negative (TN).

**Conclusions:**

The pathological subtypes of breast cancer are clearly different in metastatic behavior with regard to the sites of distant metastasis, emphasizing that this knowledge may help to determine the appropriate strategy for follow-up and guide personalized medicine.

## INTRODUCTION

Breast cancer is the most common malignancy in women worldwide [1]. Although the prognosis of breast cancer patients is generally favorable due to early detection and the comprehensive treatment, 20%–30% of patients will still develop distant metastases and cases with progressive stage only have a median two-year survival time [2–4]. The distant organs to which breast cancer preferentially metastasizes, of which bone, liver, lung and brain are among the most common sites, are associated with the patients’ survival outcome [5, 6].

Breast cancer is widely recognized as a heterogeneous disease in the sense of both primary tumor metastatic capacity and time to metastatic spread of disease. Besides common risk factors influencing the metastases processes of breast cancer largely include tumor size, histologic grade, nodal stage and receptor status [7, 8], the propensity of breast cancer to give rise to distant metastases depends on the molecular type of breast cancer. Molecular subtypes in breast cancer are first described by Perou et al. [9] according to a specific gene expression pattern and divided into four simple subtypes based on hormone receptor (HR) and human epidermal growth factor receptor 2 (HER2) status: HR+/HER2−, HR+/HER2+, HR−/HER+, and triple negative (TN). Later studies report the subtypes (BCS) are increasingly recognized to have the differences in prognosis and adjuvant therapy response [10–12]. Recently, the subtypes defined by gene expression arrays and immunohistochemistry-based subtypes are identified to concern differences in specific sites of distant metastasis [13–16]; however, data are limited and contradictory owing to insufficient sample size and heterogeneity.

In this study, we made an attempt to explore the possible relationship between the clinicopathologic factors of the primary tumor and the common sites of distant metastases in a large cohort of patients with advanced breast cancer to guide individualized patient management.

## RESULTS

### Patient characteristics and metastasis pattern

The study groups consisted of 243,896 patients, including 226,451 cases in control groups and 17,445 cases with distant metastasis. Bone metastasis was found in 8848 cases, compared with 1,000 brain metastasis cases, 3434 liver metastasis cases and 4167 lung metastasis cases. We excluded 22 patients whose survival times were classified as unknown from the analysis and 6351 cases with unknown metastasis patterns.

Supplementry Table 1 shows clinicopathological data of the patients within single metastasis sites. In all, patients diagnosed with distant metastasis were more likely to be black, lower in grade, less in duct carcinoma (DC), larger in size, have more lymph node metastasis, be found in paired or bilateral laterality and be HER2 positive (each *p* < 0.05). In patients with brain or liver metastasis, age < 65 years entirely account for 64.4%, suggesting that those are younger than patients in the control group. While age ≥ 65 accounts for 47.4 % in lung metastasis subgroup. Further, estrogen receptor (ER) positive is more commonly found in bone metastasis subgroup, whereas ER negative is extremely common in other subgroups. Similarly, progesterone receptor (PR) negative is more general present in brain, liver and lung metastasis subgroups. As expected, patients with distant metastasis are less receiving local operation and radiation compared with those in the control group.

Supplementry Table 2 shows clinicopathological data of the patients within multiple metastasis sites. Similarly, the clinicopathological characteristics of patients diagnosed with multiple metastasis insisted on the analogy with those with single metastasis. In patients with three or four metastasis sites, age < 65 years separately account for 65.6% and 73.3%, suggesting that the young are more likely to occur multiple metastasis sites. Further, estrogen receptor (ER) negative as well as progesterone receptor (PR) negative are extremely common in multiple metastasis groups. However, HER2 positive is more general present in multiple metastasis groups. As expected, patients with double or three metastasis sites are less receiving surgical operation and radiation compared with those in the control group. Nevertheless, patients with four metastasis sites are more undergoing radiotherapy compared with those in the control group.

### Association of breast cancer subtypes with the sites of distant metastases

**Figure 1:**
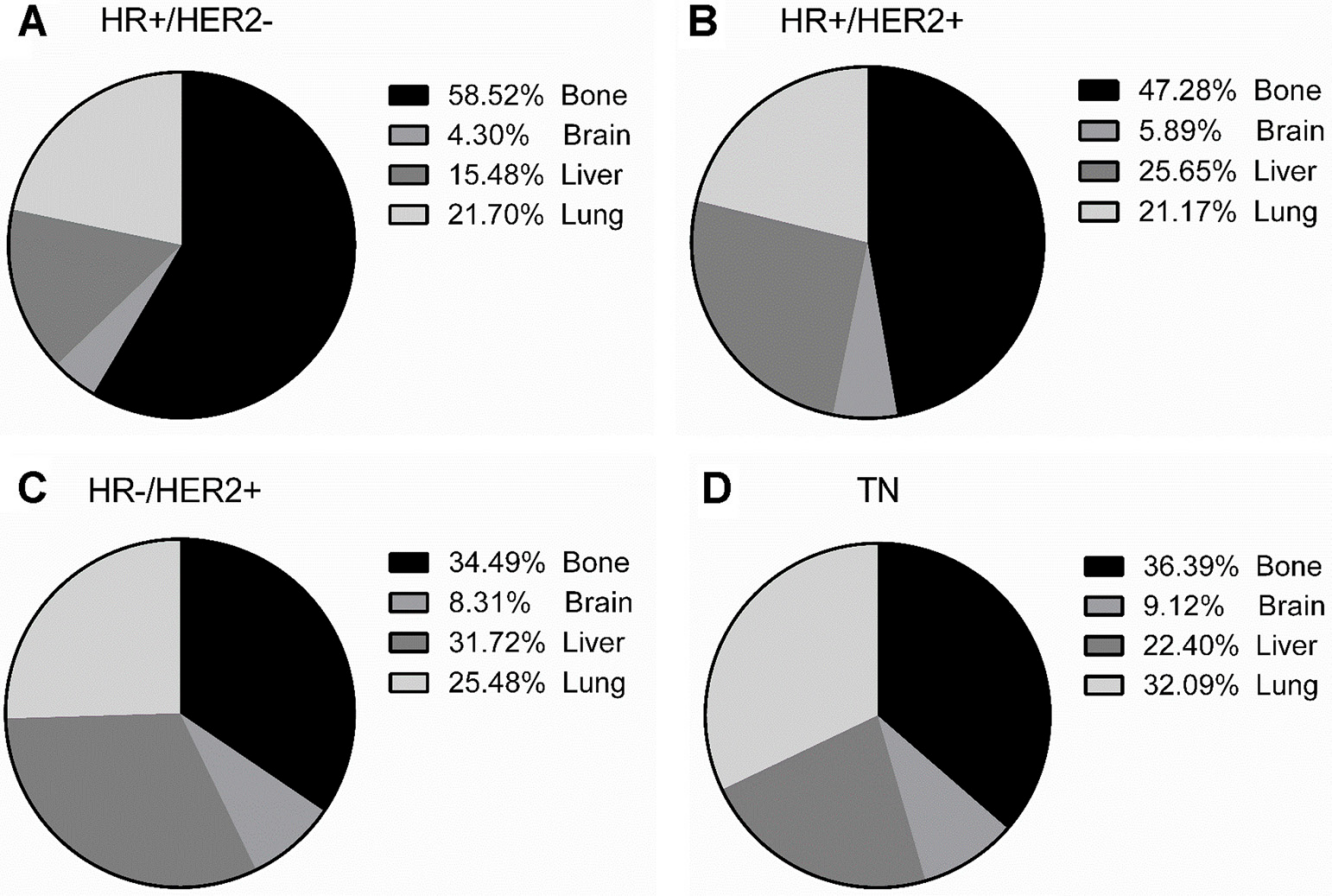
The frequencies of breast cancer subtypes at the sites of distant metastasis *HR: hormone receptor, HER2: human epidermal growth factor receptor 2, TN: triple negative*.

**Figure 2:**
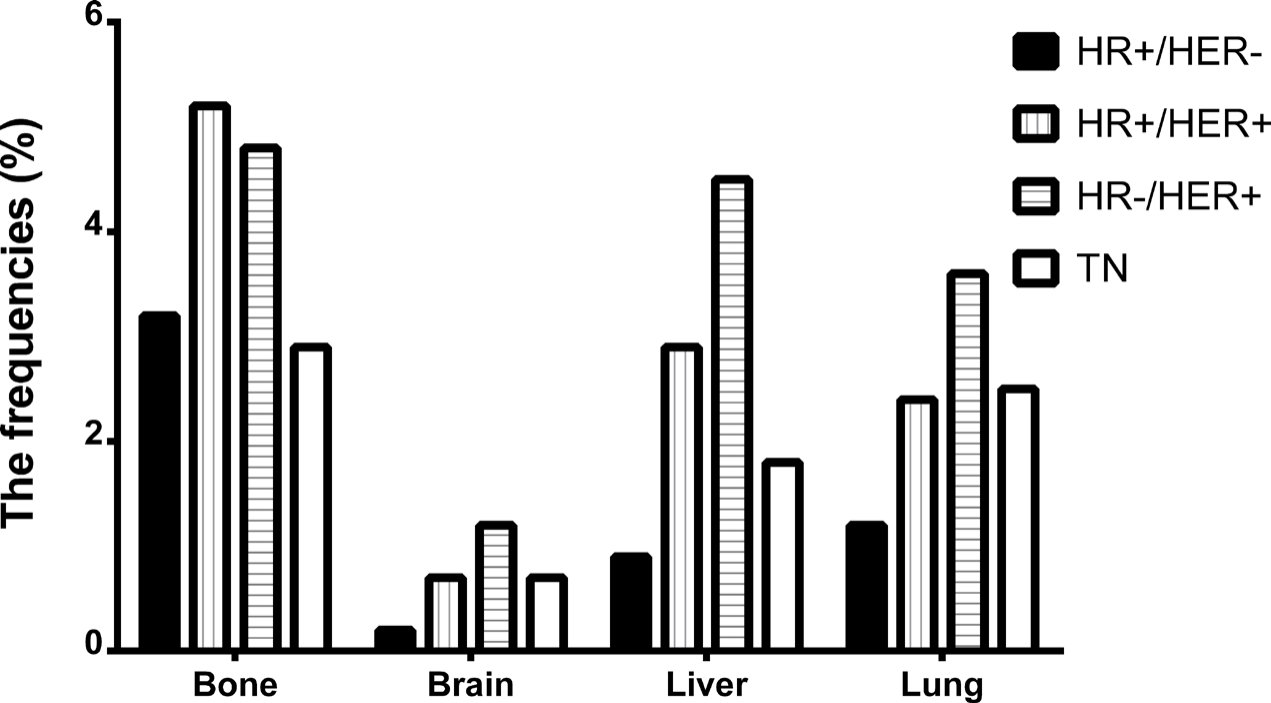
The frequencies of distant metastasis sites involvement by each breast cancer subtypes *HR: hormone receptor, HER2: human epidermal growth factor receptor 2, TN: triple negative*.

The univariate analysis revealed that patients with HR+/HER2- subtype mainly occurred bone metastasis accounting for 58.52% and the incidence reached 3.1%; HR+/HER2+ subtype patients had a high probability of bone metastasis (47.28%) and the incidence achieved 5.1%, but the proportions of liver metastasis dramatically rose; HR−/HER2+ subtype patients had a considerably high proportion of liver metastasis (31.72%) and the metastasis rates separately were up to 4.2 %; patients with TN primarily presented lung metastasis (32.09%) expect for bone metastasis and the incidence reached up to 0.7% (Table 1; Figure 1). Multivariate analysis showed that HR−/HER2+ subtypes patients had a higher probability of bone (OR = 2.442) and brain (OR = 1.978) metastasis compared to HR+/HER2− subtypes patients. While the probability of bone and liver metastases of the TN subtypes was lower than that of the HR−/HER2+ subtypes (OR = 0.771, OR = 0.510), and TN had a meaningfully higher probability of liver metastasis than HR+/HER2− subtypes (OR = 1.697) (Table 1; Figure 2).

**Table 1:** The specific pathological subtypes of breast cancer associated with the sites of distant metastasis

Site of distant metastasis/subtype	Rate (E/C,%)	Univariate		Multivariate	
		OR (95% CI)	*P*-value	OR (95% CI)	*P*-value
**Bone**					
**HR+/HER2+ vs. HR+/HER2−**	5.1/3.1	1.679 (1.572,1.793)	***P* < 0.001**	1.977 (0.988,1.174)	0.102
**HR−/HER2+ vs. HR+/HER2−**	4.6/3.1	1.548 (1.402,1.709)	***P* < 0.001**	2.442 (0.656,0.847)	**0.037**
**TN vs. HR+/HER2−**	2.8/3.1	0.904 (0.835,0.979)	**0.013**	1.049 (0.494,0.608)	0.668
**HR−/HER2+ vs. HR+/HER2+**	4.6/5.1	0.922 (0.824,1.032)	0.156	0.902 (0.58,1.402)	0.646
**TN vs. HR+/HER2+**	2.8/5.1	0.538 (0.489,0.592)	***P* < 0.001**	0.700 (0.453,1.084)	0.11
**TN vs. HR−/HER2+**	2.8/4.6	0.584 (0.518,0.659)	***P* < 0.001**	0.771 (0.671,0.886)	***P* < 0.001**
**Brain**					
**HR+/HER2+ vs. HR+/HER2−**	0.6/0.2	2.852 (2.349,3.461)	***P* < 0.001**	1.562 (0.072,1.977)	0.053
**HR−/HER2+ vs. HR+/HER2−**	1.1/0.2	5.085 (4.101,6.306)	***P* < 0.001**	1.978 (1.501,2.607)	0.010
**TN vs. HR+/HER2−**	0.7/0.2	3.088 (2.582,3.693)	***P* < 0.001**	1.358 (0.49,1.711)	0.158
**HR−/HER2+ vs. HR+/HER2+**	1.1/0.6	1.783 (1.387,2.293)	***P* < 0.001**	0.892 (0.048,6.924)	0.815
**TN vs. HR+/HER2+**	0.7/0.6	1.083 (0.868,1.351)	0.481	0.708 (0.273,1.836)	0.477
**TN vs. HR−/HER2+**	0.7/1.1	0.607 (0.478,0.772)	***P* < 0.001**	0.764 (0.583,1.002)	0.051
**Liver**					
**HR+/HER2+ vs. HR+/HER2−**	2.8/0.8	3.443 (3.127,3.792)	***P* < 0.001**	2.026 (0.386,1.963)	0.793
**HR−/HER2+ vs. HR+/HER2−**	4.2/0.8	5.382 (4.81,6.022)	***P* < 0.001**	2.219 (0.269,1.455)	0.276
**TN vs. HR+/HER2−**	1.7/0.8	2.103 (1.887,2.345)	**P < 0.001**	1.697 (1.028,1.896)	**0.005**
**HR−/HER2+ vs. HR+/HER2+**	4.2/2.8	1.544 (1.368,1.764)	***P* < 0.001**	0.541 (0.35,1.838)	0.106
**TN vs. HR+/HER2+**	1.7/2.8	0.615 (0.543,0.696)	***P* < 0.001**	0.268 (0.173,0.414)	***P* < 0.001**
**TN vs. HR−/HER2+**	1.7/4.2	0.391 (0.341,0.448)	***P* < 0.001**	0.510 (0.437,0.595)	***P* < 0.001**
**Lung**					
**HR+/HER2+ vs. HR+/HER2−**	2.3/1.2	2.028 (1.837,2.239)	***P* < 0.001**	1.591 (0.25,1.397)	0.231
**HR−/HER2+ vs. HR+/HER2−**	3.4/1.2	3.086 (2.74,3.475)	***P* < 0.001**	1.600 (0.247,1.461)	0.261
**TN vs. HR+/HER2−**	2.5/1.2	1.507 (1.31,1.735)	***P* < 0.001**	2.144 (0.896,2.641)	0.281
**HR−/HER2+ vs. HR+/HER2+**	3.4/2.3	1.080 (0.959,1.216)	0.202	1.601 (0.880,2.912)	0.123
**TN vs. HR+/HER2+**	2.5/2.3	1.06 (0.942,1.194)	0.332	1.542 (0.852,2.791)	0.152
**TN vs. HR−/HER2+**	2.5/3.4	0.717 (0.626,0.82)	***P* < 0.001**	0.945 (0.811,1.101)	0.469

*P* values calculated by logistic regression; Bold if statistically significant, *P* < 0.05.

Rate, the metastasis rate; E/C, Experiment group/Control group; HR, hormone receptor; HER2, human epidermal growth factor receptor 2; OR, odds ratio; CI, confidence interval. The multivariate model included age, sex, race, grade, histology, tumor size, node stage, ER, PR, HER2, Breast subtype, laterality, radiotherapy, local treatment.

In terms of bone metastases, the results showed that the incidence of bone metastasis in HR+/HER2+ subtypes was up to 5.1% and was significantly higher than that of the other subtypes, while there was no significant difference in the probability of bone metastasis between HR+/HER2− and HR+/HER2+. The probability of brain metastasis of HR−/HER2+ was significantly higher than HR+/HER2− subtypes, but there were no significant differences compared with HR+/HER2+ and TN subtypes. Liver metastasis was more frequently observed in the HER2 positive subtypes compared with HER2 negative subtypes by univariate and multivariate analyses. There was an increased risk associated with the HR−/HER2+ subtype compared with the HR+/HER2+ subtype, but no statistical significance was reached. In addition, it made no difference in the probability of lung metastases of four subtypes (Table 1; Figure 2).

## DISCUSSION

In this large population-based cohort of cases diagnosed with metastatic breast cancer, this study has demonstrated that various breast cancer subtypes show a strong correlation to site-specific metastasis patterns; patients with all subtypes were most prone to bone metastases, and HR−/HER2+ subtype patients had a higher probability of brain metastasis. Further, liver metastasis was more frequently observed in the HER2 positive subtypes compared with HER2 negative subtypes. While patients with TN primarily presented lung metastasis.

Bone metastasis is the most universal metastasis of breast cancer. In agreement with some prior studies, we found that HR-positive breast cancers had a propensity to give rise to bone metastases [3, 17, 18]. As the important finding, elevated expressions of cyclooxygenase 2 (COX-2) cancers tend to home into the bone, and COX-2 expression is associated with a large tumor size, a high histological grade, a negative HR status, a high cell proliferation rate, high p53 expression, presence of HER-2 amplification, and poor survival [19]. Therefore, several findings suggest that COX-2 inhibitors could reduce the risk of bone metastases in stage II and III breast cancer [20]. In addition, SNAI1 is a zinc finger transcriptional repressor of CDH1, which encodes E-cadherin. Downregulation of E-cadherin is crucial to the dissemination and invasion of cancer cells, which might augment breast cancer metastasis into the bone [21]. Likewise, the introduction of bisphosphonates is of greatest importance for the treatment of bone metastases in breast cancer, and some studies suggest that their effect could not only be restricted to bone and but also decrease relapse in other sites [22]. Thus, the impact of bisphosphonates and COX-2 inhibitors in adjuvant therapy and their effect on sites of metastases await further confirmation in the future.

Brain metastasis is infrequent as the first site of distant recurrence in the present series, but an increasing rate of brain metastasis is reported in recent years [23]. In keeping with previous studies, both the HER2 and TN subtypes are significantly associated with brain metastasis [13, 24–26]. Brain metastases are associated most frequently with high expression of nestin, prominin-1, or CK-5, but low expressed ER or PR [27]. Similarly, the WNT pathway was associated with patients relapsing to brain. The results suggest that active WNT/β-catenin signaling contributes basal breast tumors metastasizing to the brain [13]. The biological mechanisms associated with these proteins and brain metastases remain hypothetical in breast cancer, but those breast cancer subtypes might highly express certain proteins to adapt to the brain microenvironment to initiate brain metastases. Simultaneously, the metastasis site may have molecular alterations. It is demonstrated that HER2 gene in metastasis sites are acquired in approximately 20% of HER2-negative primary cases [28]. Therefore, further clinical implications for patients with breast cancer and support comprehensive profiling of metastases to inform clinical care.

Liver metastasis is the second most common pattern of breast cancer metastatic involvement. An early study reveals a trend of association with the HER2 subtypes and a tendency for fewer liver-targeting events in patients with the luminal B subtypes [13]. And HER2 subtypes shows a similar incidence rate as the luminal B subtypes [3]. These findings are consisted with the observations in our study. In addition, our study indicates that the HR−/HER2+ subtype is significantly associated with liver metastases compared with TN subtypes. The mechanisms by which the HER2-rich tumors tend to increase the risk of liver metastases remain to be elucidated. CXCR4 has been proven to be involved in promoting the invasion of these cells to internal organs, and activated HER2 could enhance the expression and function of CXCR4 [29]. Thus, further investigation into the molecular mechanisms of this relationship may provide substantial clinical utility.

For the lung metastasis patients, it demonstrates that HR+/HER2− breast cancers rarely give rise to lung metastasis compared with TN and the HR−/HER2+ subtypes in this study and HER2 positive subtypes is prone to lung metastasis compared with HER2 negative subtypes. Largely in consonance with these observations, lung metastases are found more than expected in the basal subtypes by gene expression analysis [13], while another study reveals that luminal-A subtypes exhibited lower rates of lung relapse compared with other three subtypes by tissue microarray analysis [3]. In recent studies, the focal adhesion signaling is recognized as an important modulator of organ-specific relapse, and many focal adhesion genes are up-regulated in the luminal-A subtypes and down-regulated in tumors from patients who had a lung relapse. Further, the high frequency of extracellular matrix genes that are found significantly differentially regulated can create a specific microenvironment surrounding the metastasizing cells, necessary for invading and proliferating in lung tissue [13, 30, 31]. In addition, the strongest correlation is confirmed between the EGFR-positive breast tumor and lung metastases, and as many as 75.8% of all those patients whose first distant recurrence was in the lung had either EGFR-positive or HER2-positive [27]. And EGFR is important for tumor cell motility and invasion, and HER2 for tumor cell intravasation *in vivo* experiments [32]. It should be hypothesized that clinical testing of expression of EGFR and HER2 or genomic markers may provide complementary information for predicting lung metastasis, and some inhibitors, such as lapatinib, might be of particular effect in the relatively subset of breast cancer patients who first recurs in the lungs.

Related to therapy strategies as chemotherapy, the dynamic variability or heterogeneity of cell populations provides the driving force for tumors in order to utilize selection pressures to evolve. Such the dynamic variability before and after adjuvant therapy may be the major factor in therapy failures and tumor recurrence. This study supports the novel concept that chemotherapy may reduce mutation frequency in patients with breast cancer. In addition, loss of TP53 and PIK3CA mutations may be favorable prognostic factors [33]. Moreover, disseminated tumor cells and metastatic lesions can be found throughout the body, thus considerations of intra-tumor phenotypic heterogeneity should not be limited to primary tumors. Multiple studies have reveals that the metastatic lesions contain additional mutations, and CTNNA1 was significantly enriched for mutations [34]. Further, the status of ER, PR, HER2 and Ki-67 expressions have significance in subtypes of breast cancer and determine the strategies of adjuvant therapy. Mounting data over recent year have indicated the change of ER or HER2 status are not found after neoadjuvant chemotherapy (NAC), but there is a significant difference was found in PR status [35]. And the decreasing of Ki-67 after NAC could independently predict the prognosis in patients of Luminal B, TN, and HER2 subtypes [36]. Given the clinical consequences of discordance, it urgently requires to deeply understand the differences between primary and metastatic tumors and develops the proper management of cancer patients.

The main limitations were heterogeneous population and retrospective setting for our study. The information on systemic therapy and margin control was insufficient. In doing so, HER2 targeted therapy and novel adjuvant hormone therapy remained fully utilized to significantly improve the survival. The sites of distant metastases including bone, brain, lung and liver are recorded in SEER database after 2010, but other metastatic sites were not recorded in detail. Despite the limitations, our study demonstrates that the pathological subtypes of breast cancer are clearly different in metastatic behavior with regard to the sites of distant metastasis. These observations have the potential to improve patient management and survival. A conceptual framework of the biology of breast cancer metastases needs to further develop to predict which patients are at high risk to later develop metastatic breast cancer and pursue personalized medicine.

## MATERIALS AND METHODS

### Data source and study design

We obtained data from the National Cancer Institute's Surveillance, Epidemiology, and End Results (SEER) program between 2010 and 2013. HER2 status and the sites of distant metastases were started collecting by SEER in 2010. Therefore, we used the year as the starting point. We extracted data for all cases of invasive BC diagnosed with known HR status, HER2 status and breast subtypes. That patients diagnosed with unknown subtypes were excluded. The patients without distant metastases were selected as the control group.

Demographic variables included age at diagnosis (< 35, 35–49, 50–64, > 65 years) and race (white, black, other). The cancer characteristics included grade (well differentiated, moderately differentiated, poorly differentiated, undifferentiated, unknown), tumor size (≤ 10, 10–20, 20–50, > 50 mm), N stage (N0, N1, N2, N3, NX, NA), laterality (right, left, paired, bilateral, unknown), and HR and HER2 status (positive, negative, unknown). Treatment characteristics included receipt of radiotherapy (no, yes, unknown). Patients were categorized as receiving BCS (surgery of primary site variable values of 20–24) and mastectomy (surgery of primary site variable values of 30–80). The subtypes were characterized according to the breast subtypes variable as HR+/HER2−, HR+/HER2+, HR−/HER2+ and TN.

### Statistical analysis

Patient demographics and cancer- and treatment-related characteristics were compared within subtypes using Chi square or Fisher's exact tests as appropriate. Within each variable, patients with unknown data were excluded from the comparative analysis. A matched subtype analysis was performed. The association of clinicopathologic factors and the sites of distant metastases was modeled with logistic regression analysis. Both univariate and multivariate odds ratios (ORs) and 95% confidence intervals (CIs) were calculated for each model. Predictive factors for distant metastasis were determined by multivariable logistic regression analysis, in which factors that were statistically significant in the univariate analysis were entered into the multivariable logistic regression analysis. All statistical analyses were performed using SPSS 19.0 (IBM Corporation, Armonk, NY), and all charts of frequency were prepared using GraphPad Prism 6.0. Two-sided *p* values less than 0.05 were considered statistically significant.

## SUPPLEMENTARY MATERIALS TABLES




