# Supplementary material for: Breast cancer subtypes predict the preferential site of distant metastases: a SEER based study
Source: Oncotarget. 2017 Mar 2;8(17):27990–6. doi: 10.18632/oncotarget.15856 (PMC5438624; doi:10.18632/oncotarget.15856)
Supplement: Supplementary file 1 [file oncotarget-08-27990-s001.pdf]

Breast cancer subtypes predict the preferential site of distant metastases: a SEER based study

Supplementary Materials

Supplementry Table 1: Clinical features and single metastasis sites. See Supplementry_Table_1

Supplementry Table 2: Clinical features and multiple metastasis sites. See Supplementry_Table_2
