# Supplementary material for: Breast cancer subtypes predict the preferential site of distant metastases: a SEER based study
Source: Oncotarget. 2017 Mar 2;8(17):27990–6. doi: 10.18632/oncotarget.15856 (PMC5438624; doi:10.18632/oncotarget.15856)
Supplement: Supplementary file 2 [file oncotarget-08-27990-s002.docx]

**Table 1.** Clinical features and single metastasis sites.

| **Variables** | **Control**  **N=226451(%)** | **Bone N=8844(%)** | **Brain**  **N=1000(%)** | **Liver**  **N=3434 (%)** | **Lung**  **N=4167 (%)** |
| --- | --- | --- | --- | --- | --- |
| **Survival (months)** | 21.73±13.75 | 14.66±12.83 | 9.62±10.96 | 11.30±11.71 | 12.48±12.23 |
| **Age at diagnosis, y** |  | **P< 0.001** | **P< 0.001** | **P< 0.001** | **P< 0.001** |
| **＜35** | 4063(1.8) | 218(2.5) | 24(2.4) | 134(3.9) | 81(1.9) |
| **35-49** | 42045(18.6) | 1479(16.7) | 175(17.5) | 661(19.2) | 559(13.4) |
| **50-64** | 84806(37.5) | 3461(39.1) | 445(44.5) | 1415(41.2) | 1551(37.2) |
| **≥65** | 95537(42.2) | 3686(41.7) | 356(35.6) | 1224(35.6) | 1976(47.4) |
| **Sex** |  | **P< 0.001** | 0.114 | 0.31 | **P< 0.001** |
| **Female** | 224723(99.2) | 8741(98.8) | 988(98.8) | 3413(99.4) | 4104(98.5) |
| **Male** | 1728(0.8) | 103(1.2) | 12(1.2) | 21(0.6) | 63(1.5) |
| **Race** |  | **P< 0.001** | **P< 0.001** | **P< 0.001** | **P< 0.001** |
| **white** | 180847(79.9) | 6875(77.7) | 746(74.6) | 2562(74.6) | 3063(73.5) |
| **Black** | 24180(10.7) | 1342(15.2) | 194(19.4) | 616(17.9) | 783(18.8) |
| **Other** | 20410(9.0) | 603(6.8) | 59(5.9) | 247(7.2) | 311(7.5) |
| **Unknown** | 1014(0.4) | 24(0.3) | 1(0.1) | 9(0.3) | 10(0.2) |
| **Grade** |  | **P< 0.001** | **P< 0.001** | **P< 0.001** | **P< 0.001** |
| **Well** | 50583(22.3) | 610(6.9) | 33(3.3) | 139(4.0) | 177(4.2) |
| **Moderately** | 94521(41.7) | 3091(35.0) | 274(27.4) | 935(27.2) | 1181(28.3) |
| **Poorly** | 68079(30.1) | 2774(31.4) | 375(37.5) | 1430(41.6) | 1664(39.9) |
| **Undifferentiated** | 981(0.4) | 54(0.6) | 13(2.3) | 31(0.9) | 46(1.1) |
| **Unknown** | 12287(5.4) | 2315(26.2) | 305(30.5) | 899(26.2) | 1099(26.4) |
| **Histology** |  | **P< 0.001** | **P< 0.001** | **P< 0.001** | **P< 0.001** |
| **DC** | 169011(74.6) | 5540(62.6) | 624(62.4) | 2307(67.2) | 2837(68.1) |
| **LC** | 20662(9.1) | 1124(12.7) | 53(5.3) | 240(7.0) | 168(4.0) |
| **DC and LC** | 12568(5.5) | 391(4.4) | 23(2.3) | 103(3.0) | 98(2.4) |
| **Other** | 24210(10.7) | 1789(20.2) | 300(30.0) | 784(22.8) | 104(25.5) |
| **Tumor size(mm)** |  | **P< 0.001** | **P< 0.001** | **P< 0.001** | **P< 0.001** |
| **≤ 10** | 58052(25.6) | 527(6.0) | 78(7.8) | 173(5.0) | 199(4.8) |
| **10-20** | 67960(30.0) | 728(8.2) | 85(8.5) | 275(8.0) | 268(6.4) |
| **20-50** | 73888(32.6) | 2963(33.5) | 275(27.5) | 1097(31.9) | 1280(30.7) |
| **> 50** | 19459(8.6) | 2549(28.8) | 291(29.1) | 1051(30.6 | 1445(34.7) |
| **Unknown** | 7092(3.1) | 2077(23.5) | 271(27.1) | 838(24.4) | 975(23.4) |
| **Node stage** |  | **P< 0.001** | **P< 0.001** | **P< 0.001** | **P< 0.001** |
| **N0** | 158994(70.2) | 2167(24.5) | 250(15.0) | 800(23.3) | 938(22.5) |
| **N1** | 48318(21.3) | 3554(40.2) | 377(37.7) | 1442(42.0) | 1746(41.9) |
| **N2** | 11050(4.9) | 889(10.1) | 93(9.3) | 330(9.6) | 433(10.4) |
| **N3** | 6227(2.7) | 1077(12.2) | 130(13.0) | 368(10.7) | 486(11.7) |
| **NX** | 1591(0.7) | 1151(13.0) | 149(14.9) | 490(14.3) | 552(13.2) |
| **NA** | 271(0.1) | 6(0.1) | 1(0.1) | 4(0.1) | 12(0.3) |
| **ER** |  | **P< 0.001** | **P< 0.001** | **P< 0.001** | **P< 0.001** |
| **Negative** | 36700(16.2) | 1315(14.9) | 331(33.1) | 972(28.3) | 1080(25.9) |
| **Positive** | 182683(80.7) | 6679(75.5) | 542(54.2) | 2041(59.4) | 2548(61.1) |
| **Unknown** | 7068(3.1) | 850(9.6) | 127(12.7) | 421(12.3) | 539(12.9) |
| **PR** |  | **P< 0.001** | **P< 0.001** | **P< 0.001** | **P< 0.001** |
| **Negative** | 60010(26.5) | 2511(28.4) | 471(47.1) | 1446(42.1) | 1548(37.1) |
| **Positive** | 158294(69.9) | 5385(60.9) | 387(38.7) | 1526(44.4) | 2030(48.7) |
| **Unknown** | 8147(3.6) | 948(10.7) | 142(14.2) | 462(13.5) | 589(14.1) |
| **HER2** |  | **P< 0.001** | **P< 0.001** | **P< 0.001** | **P< 0.001** |
| **Negative** | 179741(79.4) | 5868(66.4) | 558(55.8) | 1809(52.7) | 2539(60.9) |
| **Positive** | 29615(13.1) | 1599(18.1) | 255(25.5) | 1044(30.4) | 853(20.5) |
| **Unknown** | 17095(7.5) | 1377(15.6) | 187(18.7) | 581(16.9) | 775(18.6) |
| **Subtype** |  | **P< 0.001** | **P< 0.001** | **P< 0.001** | **P< 0.001** |
| **HR+/HER-** | 155452(68.6) | 5095(57.6) | 374(37.4) | 1348(39.3) | 1889(45.3) |
| **HR+/HER+** | 20697(9.1) | 1139(12.9) | 142(14.2) | 618(18.0) | 510(12.2) |
| **HR-/HER+** | 8828(3.9) | 448(5.1) | 108(10.8) | 412(12.0) | 331(7.9) |
| **TN** | 23960(10.6) | 710(8.0) | 178(17.8) | 437(12.7) | 626(15.0) |
| **Unknown** | 17514(28.4) | 1452(16.4) | 198(19.8) | 619(18.0) | 811(19.5) |
| **Laterality** |  | **P< 0.001** | **P< 0.001** | **P< 0.001** | **P< 0.001** |
| **Left** | 114938(50.8) | 4261(48.2) | 473(47.3) | 1662(48.4) | 2022(48.5) |
| **Right** | 111333(49.2) | 4099(46.3) | 452(45.1) | 1575(45.9) | 1947(46.6) |
| **Paired** | 116(0.1) | 383(4.3) | 58(5.8) | 163(4.7) | 155(3.7) |
| **Bilateral** | 22(0) | 66(0.7) | 11(1.1) | 25(0.7) | 32(0.8) |
| **Unknown** | 42(0) | 35(0.4) | 7(0.7) | 9(0.3) | 17(0.4) |
| **Radiotherapy** |  | **P< 0.001** | **P< 0.001** | **P< 0.001** | **P< 0.001** |
| **No** | 111570(49.3) | 5599(63.3) | 602(60.2) | 2581(75.2) | 3048(73.1) |
| **Yes** | 105553(46.6) | 3026(34.2) | 382(38.2) | 772(22.5) | 1028(24.7) |
| **Unknown** | 9328(4.1) | 219(2.5) | 16(1.6) | 81(2.4) | 91(2.2) |
| **Local treatment** |  | **P< 0.001** | **P< 0.001** | **P< 0.001** | **P< 0.001** |
| **M** | 97590(43.1) | 1646(18.6) | 92(9.2) | 540(15.7) | 719(17.3) |
| **BCS** | 116371(51.4) | 691(7.8) | 60(6.0) | 242(7.0) | 288(6.9) |
| **Unknown** | 12490(5.5) | 6507(73.6) | 848(84.8) | 2652(77.2) | 3160(75.8) |
| **Status** |  | **P< 0.001** | **P< 0.001** | **P< 0.001** | **P< 0.001** |
| **Alive** | 214230(94.6) | 5028(56.9) | 355(35.5) | 1548(45.1) | 1992(47.8) |
| **Dead** | 12221(5.4) | 3816(43.1) | 645(64.5) | 1886(54.9) | 2175(52.2) |
| **Breast cancer** | 5847(47.8) | 3267(85.6) | 545(84.5) | 1639(86.9) | 1840(84.6) |
| **Other** | 6374(52.2) | 549(14.4) | 100(15.5) | 247(13.1) | 335(15.4) |

*P values calculated by Pearson Chi squared testing; Bold if statistically significant, ^*^P < 0.05*

*y: years, DC:* *duct carcinoma, LC: lobular carcinoma, T: tumor, N: node; HR: hormone receptor, HER2: human epidermal growth factor receptor 2, TN: triple negative, mm:* *millimeter, M:* *Mastectomy, BCS: breast-conserving surgery.*

**Table 2.** Clinical features and multiple metastasis sites.

| **Variables** | **Control**  **N=226451(%)** | **Double N=2905(%)** | **Three**  **N=900(%)** | **Four**  **N=146(%)** |
| --- | --- | --- | --- | --- |
| **Survival (months)** | 21.73±13.75 | 12.78±13.77 | 9.65±10.73 | 7.20±13.75 |
| **Age at diagnosis, y** |  | **P< 0.001** | **P< 0.001** | **0.001** |
| **＜35** | 4063(1.8) | 75(2.6) | 29(3.2) | 6(4.1) |
| **35-49** | 42045(18.6) | 460(15.8) | 160(17.8) | 31(21.2) |
| **50-64** | 84806(37.5) | 11511(39.6) | 401(44.6) | 70(47.9) |
| **≥65** | 95537(42.2) | 1219(42.0) | 310(34.4) | 39(26.7) |
| **Sex** |  | **0.004** | **0.019** | 0.914 |
| **Female** | 224723(99.2) | 2869(98.8) | 887(98.6) | 145(99.3) |
| **Male** | 1728(0.8) | 36(1.2) | 13(1.4) | 1(0.7) |
| **Race** |  | **P< 0.001** | **P< 0.001** | 0.055 |
| **white** | 180847(79.9) | 2168(74.6) | 666(74.0) | 114(78.1) |
| **Black** | 24180(10.7) | 508(17.5) | 165(18.3) | 24(16.4) |
| **Other** | 20410(9.0) | 222(7.6) | 67(7.4) | 8(5.5) |
| **Unknown** | 1014(0.4) | 7(0.2) | 2(0.2) | 0(0.0) |
| **Grade** |  | **P< 0.001** | **P< 0.001** | **P< 0.001** |
| **Well** | 50583(22.3) | 146(5.0) | 30(3.3) | 4(2.7) |
| **Moderately** | 94521(41.7) | 913(31.4) | 284(31.6) | 33(22.6) |
| **Poorly** | 68079(30.1) | 1069(31.4) | 353(39.2) | 52(35.6) |
| **Undifferentiated** | 981(0.4) | 27(0.6) | 4(0.4) | 2(1.4) |
| **Unknown** | 12287(5.4) | 750(26.2) | 229(25.4) | 55(37.7) |
| **Histology** |  | **P< 0.001** | **P< 0.001** | **P< 0.001** |
| **DC** | 169011(74.6) | 1965(67.6) | 627(69.7) | 86(58.9) |
| **LC** | 20662(9.1) | 222(7.5) | 45(5.0) | 6(4.1) |
| **DC and LC** | 12568(5.5) | 99(3.4) | 17(1.9) | 3(2.1) |
| **Other** | 24210(10.7) | 619(21.3) | 211(23.4) | 51(34.9) |
| **Tumor size(mm)** |  | **P< 0.001** | **P< 0.001** | **P< 0.001** |
| **≤ 10** | 58052(25.6) | 153(5.3) | 45(5.0) | 13(8.9) |
| **10-20** | 67960(30.0) | 217(7.5) | 62(6.9) | 12(8.2) |
| **20-50** | 73888(32.6) | 911(31.4) | 276(30.7) | 28(19.2) |
| **> 50** | 19459(8.6) | 932(32.1) | 302(33.6) | 47(32.2) |
| **Unknown** | 7092(3.1) | 692(23.8) | 215(23.9) | 46(31.5) |
| **Node stage** |  | **P< 0.001** | **P< 0.001** | **P< 0.001** |
| **N0** | 158994(70.2) | 651(22.4) | 187(20.8) | 36(24.7) |
| **N1** | 48318(21.3) | 1220(42.0) | 420(46.7) | 65(44.5) |
| **N2** | 11050(4.9) | 303(10.4) | 84(9.3) | 120(8.2) |
| **N3** | 6227(2.7) | 346(11.9) | 106(11.8) | 14(9.6) |
| **NX** | 1591(0.7) | 381(13.1) | 103(11.4) | 19(13.0) |
| **NA** | 271(0.1) | 4(0.1) | 0(0.0) | 0(0.1) |
| **ER** |  | **P< 0.001** | **P< 0.001** | **P< 0.001** |
| **Negative** | 36700(16.2) | 601(20.7) | 226(25.1) | 54(37.0) |
| **Positive** | 182683(80.7) | 1980(68.2) | 580(64.4) | 77(52.7) |
| **Unknown** | 7068(3.1) | 324(11.2) | 94(10.4) | 15(10.3) |
| **PR** |  | **P< 0.001** | **P< 0.001** | **P< 0.001** |
| **Negative** | 60010(26.5) | 1013(34.9) | 344(38.2) | 72(49.3) |
| **Positive** | 158294(69.9) | 1537(52.9) | 450(50.0) | 54(37.0) |
| **Unknown** | 8147(3.6) | 355(12.2) | 106(11.8) | 20(13.7) |
| **HER2** |  | **P< 0.001** | **P< 0.001** | **P< 0.001** |
| **Negative** | 179741(79.4) | 1778(61.2) | 506(56.2) | 82(56.2) |
| **Positive** | 29615(13.1) | 647(22.3) | 245(27.2) | 40(27.4) |
| **Unknown** | 17095(7.5) | 480(16.5) | 149(16.6) | 24(16.4) |
| **Subtype** |  | **P< 0.001** | **P< 0.001** | **P< 0.001** |
| **HR+/HER-** | 155452(68.6) | 1442(49.6) | 392(43.6) | 54(37.0) |
| **HR+/HER+** | 20697(9.1) | 432(14.9) | 152(16.9) | 20(13.7) |
| **HR-/HER+** | 8828(3.9) | 208(7.2) | 90(10.0) | 18(12.3) |
| **TN** | 23960(10.6) | 320(11.0) | 105(11.7) | 28(19.2) |
| **Unknown** | 17514(28.4) | 503(17.3) | 161(17.9) | 26(17.8) |
| **Laterality** |  | **P< 0.001** | **P< 0.001** | **P< 0.001** |
| **Left** | 114938(50.8) | 1397(48.1) | 434(48.2) | 61(41.8) |
| **Right** | 111333(49.2) | 1364(47.0) | 423(47.0) | 72(49.3) |
| **Paired** | 116(0.1) | 114(3.9) | 32(3.6) | 9(6.2) |
| **Bilateral** | 22(0) | 23(0.8) | 9(1.0) | 3(2.1) |
| **Unknown** | 42(0) | 7(0.2) | 2(0.2) | 1(0.7) |
| **Radiotherapy** |  | **P< 0.001** | **P< 0.001** | **0.001** |
| **No** | 111570(49.3) | 1970(67.8) | 576(64.0) | 59(40.4) |
| **Yes** | 105553(46.6) | 871(30.0) | 314(34.9) | 87(59.6) |
| **Unknown** | 9328(4.1) | 64(2.1) | 10(1.1) | 0(0.0) |
| **Local treatment** |  | **P< 0.001** | **P< 0.001** | **P< 0.001** |
| **M** | 97590(43.1) | 420(14.5) | 79(8.8) | 4(2.7) |
| **BCS** | 116371(51.4) | 179(6.2) | 39(4.3) | 4(2.7) |
| **Unknown** | 12490(5.5) | 2306(79.4) | 782(86.9) | 138(94.5) |
| **Status** |  | **P< 0.001** | **P< 0.001** | **P< 0.001** |
| **Alive** | 214230(94.6) | 1407(48.4) | 350(38.9) | 49(33.6) |
| **Dead** | 12221(5.4) | 1498(51.6) | 550(61.1) | 97(66.4) |
| **Breast cancer** | 5847(47.8) | 1308(87.32) | 488(88.73) | 76(78.35) |
| **Other** | 6374(52.2) | 190(12.68) | 62(11.27) | 19(21.65) |

*P values calculated by Pearson Chi squared testing; Bold if statistically significant, P < 0.05.*

*Double: any metastasis in two sites; Three, any metastasis in three sites; Four, any metastasis in four sites.*

*y: years, DC:* *duct carcinoma, LC: lobular carcinoma, T: tumor, N: node; HR: hormone receptor, HER2: human epidermal growth factor receptor 2, TN: triple negative, mm:* *millimeter, M: Mastectomy, BCS: breast-conserving surgery.*
